# Resveratrol engages AMPK to attenuate ERK and mTOR signaling in sensory neurons and inhibits incision-induced acute and chronic pain

**DOI:** 10.1186/1744-8069-8-5

**Published:** 2012-01-23

**Authors:** Dipti V Tillu, Ohannes K Melemedjian, Marina N Asiedu, Ning Qu, Milena De Felice, Gregory Dussor, Theodore J Price

**Affiliations:** 1 Department of Pharmacology, University of Arizona, 1501 N Campbell Ave, PO BOX 245050, Tucson, AZ 85724, USA; 2 Graduate Interdisciplinary Program in Neuroscience, University of Arizona, Tucson, USA; 3 Bio5 Institute, University of Arizona, Tucson, USA

## Abstract

**Background:**

Despite advances in our understanding of basic mechanisms driving post-surgical pain, treating incision-induced pain remains a major clinical challenge. Moreover, surgery has been implicated as a major cause of chronic pain conditions. Hence, more efficacious treatments are needed to inhibit incision-induced pain and prevent the transition to chronic pain following surgery. We reasoned that activators of AMP-activated protein kinase (AMPK) may represent a novel treatment avenue for the local treatment of incision-induced pain because AMPK activators inhibit ERK and mTOR signaling, two important pathways involved in the sensitization of peripheral nociceptors.

**Results:**

To test this hypothesis we used a potent and efficacious activator of AMPK, resveratrol. Our results demonstrate that resveratrol profoundly inhibits ERK and mTOR signaling in sensory neurons in a time- and concentration-dependent fashion and that these effects are mediated by AMPK activation and independent of sirtuin activity. Interleukin-6 (IL-6) is thought to play an important role in incision-induced pain and resveratrol potently inhibited IL-6-mediated signaling to ERK in sensory neurons and blocked IL-6-mediated allodynia in vivo through a local mechanism of action. Using a model of incision-induced allodynia in mice, we further demonstrate that local injection of resveratrol around the surgical wound strongly attenuates incision-induced allodynia. Intraplantar IL-6 injection and plantar incision induces persistent nociceptive sensitization to PGE_2 _injection into the affected paw after the resolution of allodynia to the initial stimulus. We further show that resveratrol treatment at the time of IL-6 injection or plantar incision completely blocks the development of persistent nociceptive sensitization consistent with the blockade of a transition to a chronic pain state by resveratrol treatment.

**Conclusions:**

These results highlight the importance of signaling to translation control in peripheral sensitization of nociceptors and provide further evidence for activation of AMPK as a novel treatment avenue for acute and chronic pain states.

## Background

Incision associated with surgery causes acute pain and surgery has been identified as a potential major cause of chronic pain conditions [1-3]. Between 10 and 50% of patients develop chronic pain following surgical procedures such as groin hernia repair, breast and thoracic surgery, leg amputation, or coronary artery bypass surgery [2]. Despite improvements in post-surgical pain treatment strategies, the incidence of moderate to severe pain after surgery is still high in several patient populations [4,5]. Moreover, the exact mechanisms involved in the development of persistent pain following surgery have not been elucidated. Interleukin 6 (IL-6), a pro-inflammatory cytokine, is a significant mediator of nociceptive plasticity in pre-clinical pain models and is implicated in several human pain conditions. Serum IL-6 levels increase significantly in patients immediately after surgery [6-8] and circulating IL-6 levels are proportional to the extent of tissue injury during an operation, rather than being proportional to the duration of the surgical procedure itself [9]. Furthermore, IL-6 levels have been shown to be elevated in skin around incision sites [10,11] and it has been implicated in preclinical incision-induced pain models [12-14]. Although these reports are suggestive of involvement of IL-6 in post-surgical pain, the precise mechanisms by which IL-6 drives post-surgical pain are poorly understood. However, IL-6 has been implicated as an important player in many preclinical pain models and elegant genetic studies have demonstrated that IL-6's pain promoting qualities are mediated by IL-6 receptors expressed by nociceptors [15,16].

Recently we demonstrated that IL-6 causes induction of nascent protein synthesis in primary afferent neurons and their axons which can contribute to increased nociceptive sensitivity [17]. We have also shown that AMP-activated protein kinase (AMPK) activators reverse mechanical allodynia in neuropathic pain models and that these compounds negatively regulate protein synthesis in sensory afferents [18]. AMPK, the energy sensor of the cell, is a heterotrimeric Ser/Thr protein kinase activated by alterations in cellular AMP: ATP ratio. Once activated, AMPK inhibits ATP consuming anabolic processes such as protein translation [19]. AMPK activation achieves these effects largely through inhibition of mammalian target of rapamycin (mTOR) signaling [19] but AMPK activation has also been linked to inhibition of mitogen activated protein kinase (MAPK) signaling [18,20]. We hypothesized that activation of AMPK signaling pathway may represent a novel pharmacological mechanism for the treatment of post-surgical pain.

To test this hypothesis, we have utilized resveratrol, a natural polyphenol found in red grapes and wine, which has previously been shown to increase AMPK activity in neurons [21]. Although several studies originally described resveratrol as an activator of sirtuin enzymes, which are NAD-dependent deacetylases [22-25] these results have been challenged based on lack of specificity in screening assays [26,27]. Moreover, several recent in vivo studies strongly suggest that resveratrol effects are independent of sirtuins. On the other hand, resveratrol is a highly potent and efficacious activator of AMPK [28-30] and its metabolic effects are dependent on α subunit AMPK expression suggesting that AMPK is the major target for resveratrol in vivo [31]

Herein, we demonstrate that resveratrol activates AMPK and suppresses translation regulation pathways in sensory neurons in a dose-dependent, time-dependent and reversible manner. We also show that resveratrol inhibits both acute and persistent sensitization in an IL-6-induced hyperalgesic priming model as well as in a model of postsurgical pain. These findings suggest that resveratrol may have utility in the treatment of post-surgical pain and further implicate AMPK as a novel target for the development of analgesics.

## Results

### Resveratrol suppresses signaling to translation machinery in sensory neurons

**Figure 1:**
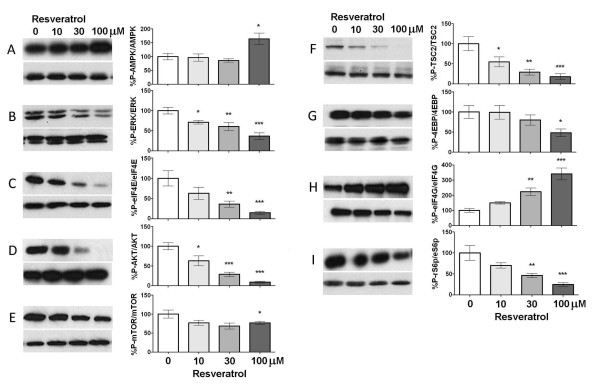
**Resveratrol suppresses ERK and mTOR signaling in sensory neurons in a concentration-dependent manner**. Treatment of TG neurons with resveratrol (10, 30, and 100 μM) for 1 h induces a concentration-dependent increase in phosphorylation of AMPK (A). Resveratrol treatment significantly decreases the phosphorylation of ERK (B), eIF4E (C), AKT (D), mTOR (E), TSC2 (F) 4EBP (G) and rS6p (I) but increases eIF4G phosphorylation (H).

While resveratrol has been shown to stimulate AMPK and inhibit mTOR signaling in cell-lines and some neural tissues, its effect on sensory neurons is unknown. Hence, we first asked whether resveratrol treatment influenced AMPK activity or signaling pathways involved in regulating cap-dependent protein translation in cultured trigeminal ganglion (TG) neurons from mice grown in the presence of NGF (50 ng/ml) for 5 days. TG cultures were treated with vehicle or increasing concentrations (10, 30 or 100 μM) of resveratrol (Figure 1) for 1 h. Resveratrol activated AMPK in a dose dependent manner (Figure 1A) and suppressed activity in signaling pathways that promote cap-dependent translation. Specifically, these changes included significantly decreased phosphorylation of extracellular signal regulated kinase (ERK, Figure 1B) and its downstream target involved in translation control eukaryotic initiation factor (eIF) 4E (Figure 1C). Resveratrol also decreased AKT (Figure 1D), mTOR (Figure 1E) and tuberin sclerosis protein 2 (TSC2, Figure 1F) phosphorylation indicating negative regulation of the mTOR pathway in TG neurons. Consistent with this notion, eIF4E binding protein (4EBP, Figure 1G) and ribosomal S6 protein (rS6p, Figure 1I), which are downstream mTOR targets, demonstrated decreased phosphorylation upon resveratrol treatment. Finally, resveratrol increased eIF4G phosphorylation(Figure 1H), an effect that can occur independently of mTOR signaling [32] and that is uncoupled from eIF4G-mediated eIF3 recruitment [33,34]. We have observed similar effects with other AMPK activators (e.g. A769662) [18]. Thus, in cultured TG neurons, resveratrol activates AMPK and suppresses signaling via the ERK and mTOR pathways to translation machinery suggesting a concentration-dependent inhibition of cap-dependent translation by resveratrol in sensory neurons.

**Figure 2:**
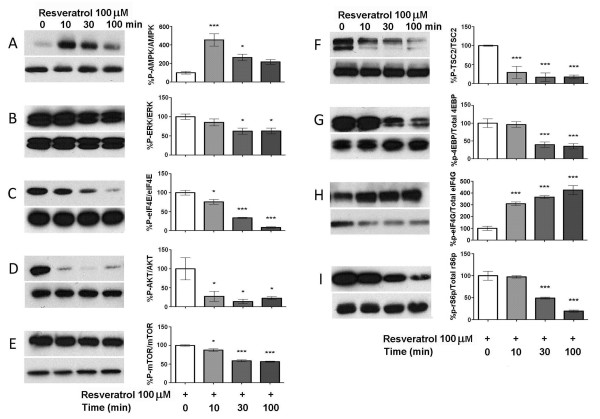
**Suppression of ERK and mTOR signaling by resveratrol is time dependent**. TG neurons were treated with 100 μM resveratrol for 0, 10, 30, and 100 min. Resveratrol induces an increase in phosphorylation of AMPK (A) maximally with 10 and 30 min treatment. Resveratrol decreases the phosphorylation of ERK (B), eIF4E (C), AKT (D), mTOR (E), TSC2 (F) 4EBP (G) and rS6 (I) and this effect is time-dependent. Resveratrol increased eIF4G phosphorylation (H).

Having established a concentration-dependent effect of resveratrol on TG neurons in culture, we next asked whether these effects were time dependent. Resveratrol, at a maximally effective dose (100 μM), was applied to TG neurons for 10, 30 or 100 min and activity in signaling pathways was assessed by Western blot (Figure 2). Resveratrol activated AMPK maximally at 10 and 30 min treatment (Figure 1A). Similarly, resveratrol suppressed activity in the ERK (Figure 2B and 2C) and mTOR pathways (Figure 2D-I) over the time course of resveratrol exposure.

**Figure 3:**
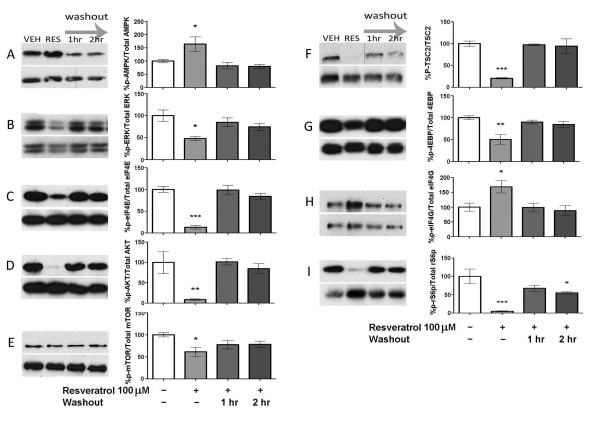
**Suppression of ERK and mTOR signaling by resveratrol is reversible**. TG neurons were treated with 100 μM resveratrol for 1 h followed by 1 or 2 h washout periods. Effects of resveratrol were reversible in all cases upon 1 h washout.

Because resveratrol led to a profound inhibition of ERK and mTOR signaling pathways, we next asked whether this effect is reversible. TG cultures were treated with resveratrol for 1 h followed by 1 or 2 h washout periods. Resveratrol led to a reversible activation of AMPK (Figure 3A) and a reversible inhibition of both ERK (Figure 3B and 3C) and mTOR signaling (Figure 3D-H). Hence, resveratrol dose- and time-dependently activates AMPK and inhibits ERK and mTOR signaling in a reversible fashion in sensory neurons.

**Figure 4:**
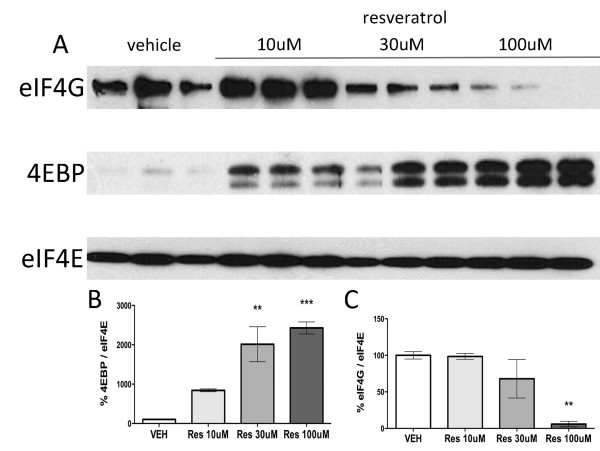
**Resveratrol suppresses eIF4F complex formation in sensory neurons**. TG neurons were treated with resveratrol (10, 30 and 100 μM) for 1 h. A) Western blot for eIF4G, 4EBP and eIF4E from trigeminal neurons co-precipitated using 7-methyl-GTP conjugated beads. B) Resveratrol induces a significant increase in 4EBP (negative regulator of translation) association with the cap-binding protein eIF4E in a dose-dependent manner. C) Resveratrol induces a significant decrease in eIF4G association with the cap-binding protein eIF4E (a component of eIF4F complex) in a dose-dependent manner.

Finally, we asked whether resveratrol treatment leads to an inhibition of cap-dependent translation in TG neurons. Cap-dependent translation requires eIF4F complex formation on the 5'cap structure of mRNAs [35] and this can be assayed with a cap pull-down assay that assesses eIF4G and 4EBP binding to eIF4E [36]. The eIF4F complex is composed of eIF4E bound to eIF4A and eIF4G whereas 4EBP binding to eIF4E is indicative of inhibition of cap-dependent translation because 4EBP represses eIF4A and 4 G binding to eIF4E [35]. Resveratrol treatment for 1 h led to a profound increase in 4EBP binding to eIF4E and a parallel decrease in eIF4G binding (Figure 4A-C). Hence, resveratrol concentration-dependently inhibits eIF4F complex formation in sensory neurons consistent with inhibition of cap-dependent translation.

### Resveratrol-mediated inhibition of ERK and mTOR does not require Sirt1

While the above results strongly suggest that resveratrol acts via activation of AMPK, there have been conflicting reports suggesting that resveratrol produces its effect by activation of Sirt1, a NAD-dependent deacetylase [22-25]. To assess whether Sirt1 may play a role in this process we utilized a Sirt1 inhibitor to ask whether it would block the effects of resveratrol on ERK and mTOR signaling. Trigeminal primary neuronal cultures were pre-treated for 1 h and then co-treated with nicotinamide (10 mM), which inhibits Sirt1, in the presence of resveratrol (100 μM) for 1 h (Table 1). Nicotinamide co-incubation had no effect on resveratrol-mediated activation of AMPK or inhibition of ERK or mTOR signaling. Likewise, if resveratrol activates Sirt1, Sirt1 activators should be able to recapitulate effects produced by resveratrol. Hence, to further rule out a role for Sirt1, TG cultures were treated with vehicle or a Sirt1 activator, CAY10602 [37] (20 and 60 μM) for 1 h. Treatment with CAY10602 did not change AMPK, mTOR or ERK levels (Table 2). These results rule out a role for Sirt1 in resveratrol mediated regulation of AMPK, ERK and mTOR signaling and support the conclusion that resveratrol engages AMPK signaling to inhibit ERK and mTOR in primary sensory neurons.

**Table 1:** Sirt1 inhibition does not reverse resveratrol-induced effects on TG neurons.

Antibody	Vehicle	nicotinamide 10 mM + resveratrol 100 μM	resveratrol 100 μM
**p-AMPK/AMPK**	100 ± 7.3	199.8 ± 28.1	222.4 ± 40.0

**p-ERK/ERK**	100 ± 11.8	41.8 ± 8.5 **	47.2 ± 8.8 **

**p-eIF4E/eIF4E**	100 ± 5.4	27.6 ± 4.7 ***	30.2 ± 6.2 ***

**p-AKT/AKT**	100 ± 6.2	11.6 ± 3.1 ***	12.5 ± 2.4 ***

**p-mTOR/mTOR**	100 ± 4.4	70.6 ± 4.4 *	62.9 ± 6.4 **

**p-TSC2/TSC2**	100 ± 8.3	26.6 ± 4.2 ***	36.5 ± 5.2 ***

**p-4EBP/4EBP**	100 ± 2.3	38.5 ± 3.0 ***	37.9 ± 7.2 ***

**p-rS6p/rS6p**	100 ± 7.0	33.9 ± 5.9 ***	36.5 ± 6.8 ***

TG neuronal cultures were pre-treated with nicotinamide or vehicle for 1 h and then co-treated with nicotinamide (10 mM), a sirt1 inhibitor, in the presence of resveratrol for 1 h. The phosphorylated levels of AMPK, ERK, eIF4E, AKT, mTOR, 4EBP and rS6p were unchanged by nicotinamide

**Table 2:** Sirt1 activators fail to recapitulate resveratrol-induced effects on TG neurons.

Antibody	Vehicle	CAY10602 20 μM	CAY10602 60 μM
**p-AMPK/AMPK**	100 ± 13.6	95.0 ± 14.4	107.3 ± 16.6

**p-ERK/ERK**	100 ± 6.50	117.2 ± 5.91	126.4 ± 3.32 *

**p-eIF4E/eIF4E**	100 ± 13.0	105.9 ± 17.1	102.0 ± 17.0

**p-AKT/AKT**	100 ± 11.8	105.8 ± 5.0	107.2 ± 7.5

**p-mTOR/mTOR**	100 ± 8.8	103.8 ± 17.0	125.7 ± 18.5

**p-TSC2/TSC2**	100 ± 3.3	97.8 ± 6.8	115.1 ± 5.2

**p-4EBP/4EBP**	100 ± 8.3	126.2 ± 11.1	122.9 ± 8.5

**p-eIF4G/eIF4G**	100 ± 14.1	76.2 ± 13.8	109.0 ± 16.7

**p-rS6p/rS6p**	100 ± 12.5	120.3 ± 6.8	134.0 ± 10.5

TG neuronal cultures were treated with the sirt1 activator, CAY10602 (20 and 60 μM) for 1 h. The phosphorylated levels of AMPK, ERK, eIF4E, AKT, mTOR, 4EBP and rS6p were not changed by CAY10602

### Resveratrol blocks IL-6-induced signaling and IL-6-mediated allodynia

**Figure 5:**
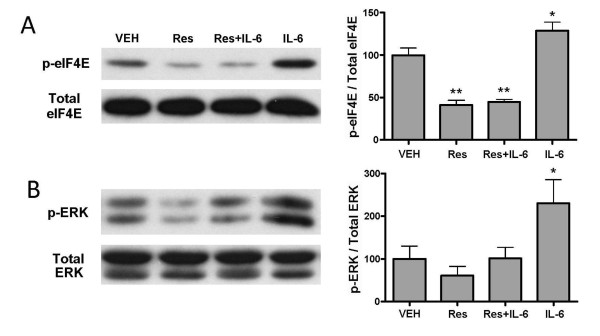
**Resveratrol blocks IL-6 induced signaling in sensory neurons**. TG neuron cultures were pre-treated with resveratrol (100 μM, 15 min) followed by subsequent co-treatment with IL-6 (50 ng/ml, 15 min). Western blot for eIF4E (A) and ERK (B) from TG neurons treated with IL6 and/or resveratrol. Resveratrol blocked IL-6 mediated phosphorylation of eIF4E and ERK in TG cultures.

Multiple lines of evidence indicate that IL-6 is an important mediator of nociceptive plasticity in postsurgical pain. While the results above demonstrate that resveratrol decreases ERK and mTOR signaling in TG neurons, it is not known whether resveratrol is capable of blocking signaling via ERK or mTOR engaged by extracellular signals. Hence, we asked whether resveratrol blocks IL-6-induced changes ERK/eIF4E signaling in primary afferent neurons [17]. Pretreatment of the TG cultures with resveratrol (100 μM, 15 min) and subsequent co-treatment with IL-6 (50 ng/ml, 15 min) completely blocked IL-6 mediated phosphorylation of ERK and eIF4E in TG cultures (Figure 5). These findings indicate that resveratrol blocks IL-6-induced signaling in sensory neurons.

**Figure 6:**
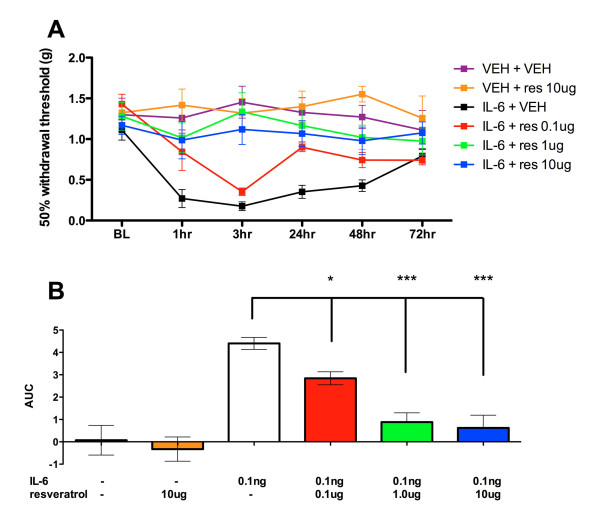
**Local resveratrol blocks IL-6 induced acute allodynia in a dose-related manner**. A) Intraplantar injection of IL-6 (0.1 ng) and co-treatment with resveratrol (0.1, 1 and 10 μg) blocks IL-6 induced allodynia. B) Area under the curve (AUC) analysis shows that resveratrol reduces IL-6 induced allodynia in a dose-related manner.

Because resveratrol inhibits IL-6-mediated ERK/eIF4E signaling in sensory neurons we hypothesized that resveratrol would inhibit IL-6-mediated allodynia in vivo. Intraplantar injection of IL-6 (0.1 ng) produces acute mechanical allodynia that lasts for ~ 3 d, with complete resolution by day 4 (Figure 6A). Co-injection with resveratrol (0.1 μg or 1 μg or 10 μg) dose-relatedly blocked IL-6-induced allodynia (Figure 6A and 6B). There was no statistically significant difference between resveratrol and vehicle treated groups. Hence, resveratrol blocks both IL-6-mediated signaling via the ERK/eIF4E pathway and IL-6-induced allodynia. These findings suggest that resveratrol may be an efficacious compound for use in pain conditions linked to IL-6 signaling, such as post-incisional pain.

### Resveratrol inhibits allodynia in a mouse model of post-surgical pain

**Figure 7:**
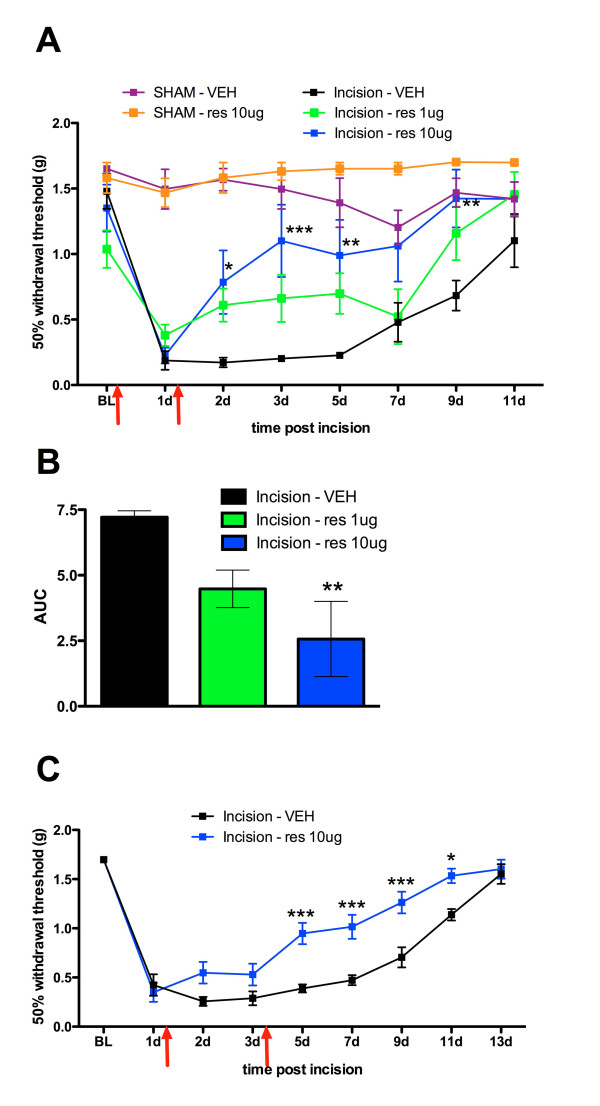
**Local resveratrol blocks plantar incision-induced allodynia in a dose-related manner**. Animals received a plantar incision on the left hindpaw. Resveratrol (1 μg or 10 μg) or vehicle was injected into the paw around the incision either immediately following incision and 1 day post incision (A, B) or 1 and 3 days following incision (C). **A) **Resveratrol injection (1 μg or 10 μg) immediately following incision and 1 day post incision significantly blocked plantar incision induced allodynia in a dose-dependent manner. B) Area under the curve (AUC) analysis showing dose-related effects in A. C) Resveratrol injection (10 μg) on day 1 and 3 following incision significantly blocks plantar incision induced allodynia. Red arrows show times of resveratrol injection.

The above results predict that resveratrol should be effective in blocking allodynia in a model of post-surgical pain. We utilized a mouse model of incisional pain to assess if resveratrol can prevent development of allodynia following the plantar incision. Animals received a plantar incision on the left hindpaw. Resveratrol (1 μg or 10 μg) or vehicle was injected into the paw around the incision either immediately following incision and 24 hrs post surgery or 1 and 3 days following incision. Mice with plantar incision that received vehicle displayed mechanical allodynia lasting for at least 9 days. In contrast, animals that received resveratrol at the time of incision and again 1 day later showed blunted allodynia and this effect was dose-related (Figure 7A and 7B). Moreover, administering resveratrol 1 and 3 days following incision significantly inhibited mechanical allodynia induced by incision (Figure 7C). No changes in threshold were observed in sham animals receiving resveratrol. These results assert that resveratrol can be a potentially efficacious treatment for post-surgical pain.

### Resveratrol blocks persistent sensitization induced by IL-6 injection and plantar incision

**Figure 8:**
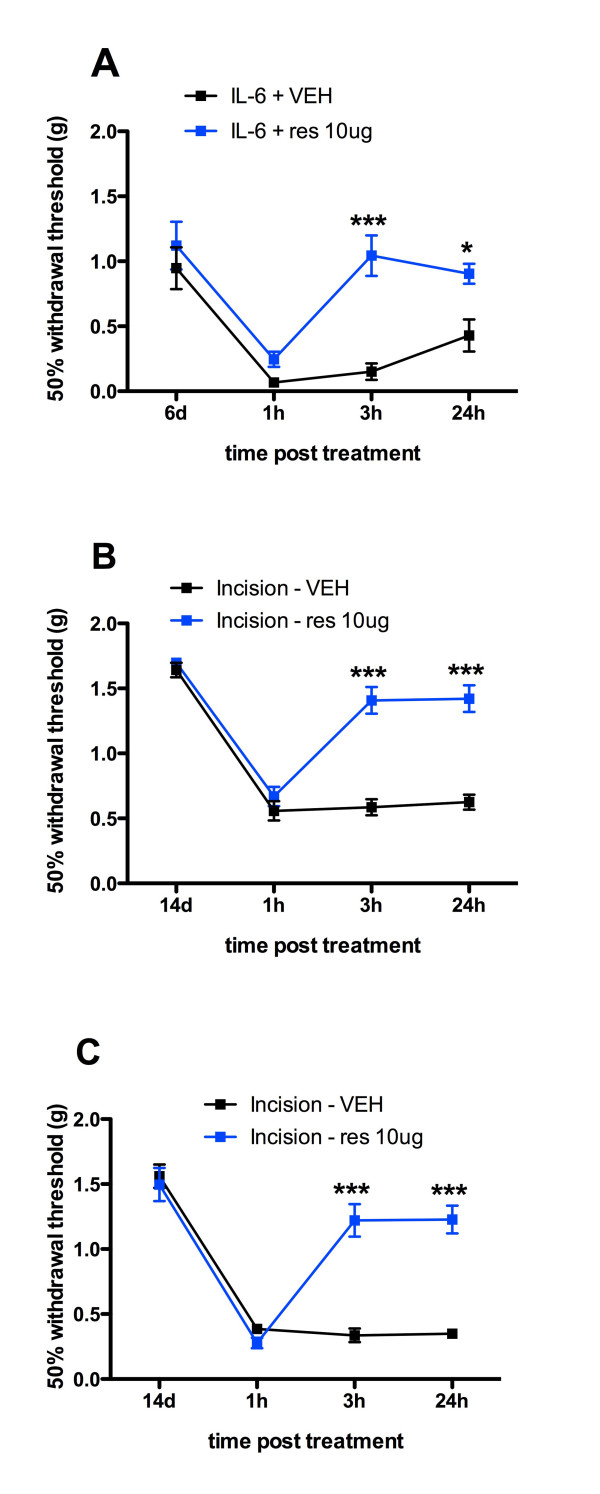
**Resveratrol blocks IL-6- and plantar incision-induced persistent sensitization**. A) Intraplantar injection of IL-6 (0.1 ng) with resveratrol (10 μg) co-treatment on Day 1 abolished the IL-6 induced persistent sensitization precipitated by PGE_2 _injection on day 6. B) Intraplantar injection of resveratrol (10 μg) at the time of incision and 1 day post incision abolished plantar incision-induced persistent sensitization precipitated by PGE_2 _injection on day 14 after incision. C) Intraplantar injection of resveratrol (10 μg) on day 1 and 3 post incision abolished plantar incision induced persistent sensitization precipitated by PGE_2 _injection on day 14 after incision.

Persistent pain is a common feature experienced by many patients undergoing surgical procedures [2]. Therefore, we assessed if resveratrol is effective in blocking persistent sensitization induced by IL-6 injection and plantar incision. Persistent sensitization can be revealed by, among other stimuli, a second intraplantar injection of inflammatory mediator, in this case PGE_2 _(100 ng), after the resolution of initial allodynia [38]. For the IL-6 induced persistent sensitization, co-treatment of resveratrol with IL-6 on Day 1 abolished the IL-6 induced persistent sensitization following PGE2 injection on day 6 (Figure 8A). Similarly, in the incision model, treatment with resveratrol at the time of incision and 1 day later or 1 and 3 days following incision both abolished persistent sensitization precipitated by PGE_2 _injection 14 days after incision. Hence, resveratrol not only inhibits allodynia induced by IL-6 or plantar incision but it also blocks the development of persistent nociceptive sensitization.

## Discussion

The present findings make a compelling case for the use of resveratrol as a local treatment for both incision induced pain and prevention of chronic pain induced by incision. They show that resveratrol potently and efficaciously inhibits ERK and mTOR signaling in sensory neurons in vitro. The mTOR [39-42] and ERK pathways [43] have been linked to pathology in multiple preclinical pain models and our previous findings strongly implicate these pathways in the induction of mechanical allodynia by IL-6 and NGF [17,44] and the development and maintenance of nerve-injury induced allodynia [18]. The pharmacological action of resveratrol observed in our in vitro experiments is linked to activation of AMPK and in vivo effects are consistent with engagement of AMPK. We have previously implicated AMPK activation in alleviation of neuropathic pain [18], hence, the findings described herein expand the potential clinical usefulness of AMPK activators into the area of post-surgical pain. We conclude that diverse pharmacological mechanisms for activation of AMPK may have utility as novel analgesics for a variety of pain conditions.

Anabolic processes, such as protein synthesis, are orchestrated by upstream kinases that signal to the translation machinery [35] such as mTOR and ERK. These kinases can be targeted individually by selective inhibitors or they can be negatively modulated by endogenous signaling factors that act on these pathways [45]. A crucial kinase for negative regulation of translation is the ubiquitous, energy-sensing kinase AMPK. Activation of AMPK by depletion of cellular nutrients or through pharmacological intervention results in a dampening of signaling to the translation machinery [45]. This is the natural cellular response to energy deprivation wherein high AMP levels signal to AMPK thereby shutting down anabolic processes when nutrient levels are low. AMPK is not solely regulated by cellular homeostatic mechanisms as it can also be targeted pharmacologically via a number of investigational compounds (e.g. AICAR and A769662 [46]), natural products (resveratrol [29,30]) and by the widely clinically available and safe drug metformin [47,48]. AMPK negatively regulates mTOR via activation of mTOR's negative regulator, TSC2 [49]. This results in a profound inhibition of mTOR and its downstream targets involved in translation control (e.g. 4EBP and ribosomal S6 kinase and rS6p [49]). Activation of AMPK also negatively regulates ERK activity induced by growth factors and cytokines [20]. This likely occurs via phosphorylation of the insulin receptor substrate 1 (IRS1) protein at Serine 789 [50]. IRS-1 is a critical component of the signaling module of all tyrosine kinase receptors (Trks) and is linked to GP130 (the IL-6 signal transduction receptor) signaling [51]. This interaction may explain the inhibitory effect of resveratrol on IL-6-mediated ERK/eIF4E signaling observed here. Hence, engaging AMPK with potent activators of this pathway (e.g. resveratrol) represents a unique opportunity to achieve inhibition of pain-related signaling because it harnesses the cell's natural mechanism for dampening signaling in two pathways strongly implicated in pain amplification in the periphery, ERK [43] and mTOR [18,39-42].

Accordingly, we hypothesized that AMPK activators may represent a novel tool for the treatment of post-surgical pain. We chose to focus on resveratrol for these experiments because resveratrol is a potent and efficacious activator of AMPK [30]. Our findings clearly demonstrate that local application of resveratrol to the site of incision reduces mechanical allodynia, and, importantly, prevents the transition to a chronic pain-like state as measured by PGE_2 _precipitated persistent nociceptive sensitization. These findings are consistent with previous experiments where we have shown that inhibition of translation regulation signaling during the initiation of allodynia induced by IL-6 or IL-6 and NGF prevents the development of persistent nociceptive sensitization, which, importantly, can be precipitated by a variety of stimuli, not solely PGE_2 _[44,52]. In fact, precipitation of persistent nociceptive sensitization following incision can be induced by administration of opioid antagonists suggesting that precipitation does not even require subsequent injury [53]. Moreover, we have recently shown that AMPK activators reduce peripheral nerve injury-induced allodynia and decrease excitability of sensory neurons in vitro [18]. While here, and in our previous work [18], we have largely ascribed the effects of AMPK activators to sensory neurons, we cannot rule out potential effects on other cell types in the behavioral effects observed. These findings collectively create a compelling case for the further exploration and development of AMPK activators for the treatment of post-surgical pain.

While the pharmacological action of resveratrol has been an area of controversy, most evidence now points to AMPK as the major target of resveratrol. As described above, much attention was originally paid to resveratrol as an activator of sirtuins, in particular sirt1. However, subsequent studies have questioned these original results and recent studies in transgenic animals point to AMPK as a requisite component of resveratrol signaling [31]. Resveratrol, stimulates AMPK in a liver kinase B1 (LKB1) -dependent fashion, similar to the upstream activation of AMPK by metformin [30,54]. We found that resveratrol stimulates AMPK in TG neurons in a concentration- and time-dependent fashion and that this AMPK activation is correlated with decreased ERK and mTOR signaling, events that we have previously shown are stimulated by other AMPK activators in TG and DRG neurons [17,18]. Inhibition or activation of sirt1 failed to inhibit or recapitulate the effects of resveratrol, respectively, ruling out an effect of sirtuins in our experiments. Several other mechanisms of action have been ascribed to resveratrol including inhibition of inducible cyclooxygenase [55] and inhibition of cyclin-dependent kinase 5 [56]. It is unlikely that these mechanisms contribute to the inhibition of ERK and mTOR signaling that we have observed in TG neurons in vitro; however, we cannot exclude the potential contribution of these effects of resveratrol to our behavioral results. Finally, resveratrol has been shown to possess voltage gated-sodium channel inhibition properties [57]. This effect has a slow onset (minutes of drug application is needed), which could be a result of slowly developing direct block of sodium channels, but is more consistent with AMPK activation. In support of the latter conclusion, we have shown that other AMPK activators induce a profound decrease in sensory neuron excitability via a suppression of ramp-current evoked spiking [18].

## Conclusion

Several previous reports have demonstrated an anti-allodynic or anti-hyperaglesic effect of resveratrol in preclinical pain models including the formalin model [58], complete Freund's adjuvant-induced inflammation [59] and nucleus pulposus-induced allodynia [60]. While, again, several mechanisms of action have been ascribed to resveratrol in these assays, our in vitro findings provide novel evidence linking resveratrol's anti-allodynic effects in the periphery to ERK and mTOR inhibition via activation of AMPK. Because resveratrol has poor bioavailability that can be sensitive to physiological factors when given systemically [61], we focused on its local effects in incision-induced pain. Resveratrol is a natural product that can be made in different preparations for human use (it is currently sold as a dietary supplement). Based on our present results, we propose that preparations of resveratrol for local use in post-surgical pain situations may be clinically useful in a similar fashion (but with obviously different mechanisms of action) to highly purified capsaicin surgical wound infusions [62]. Such preparations may afford inhibition of nociceptor sensitization and protect against a transition to chronic pain induced by surgery.

## Materials and methods

### Experimental animals

Male ICR mice (Harlan, 20-25 g) were used for the study. All animal procedures were approved by the Institutional Animal Care and Use Committee of The University of Arizona and were in accordance with International Association for the Study of Pain guidelines.

### Behavior testing

For the testing, animals were placed in acrylic boxes with wire mesh floors and allowed to habituate for approximately 1 h on all testing days. Paw withdrawal thresholds were measured using calibrated von Frey filaments (Stoelting, Wood Dale, IL) by stimulating the plantar aspect of left hind paw using the up-down method [63].

### IL-6 priming and behavior testing

A mouse model for 'hyperalgesic priming' originally developed by Levine and colleagues [for review see [52]] and adapted for mice [38] was used for the study. Baseline mechanical thresholds of the left hind paw were measured prior to IL-6 injection. For acute sensitization experiments, IL-6 (0.1 ng) was injected into the plantar surface of the left hind paw in a volume of 25 μl (diluted in saline). Resveratrol (0.1, 1, or 10 μg) or vehicle was co-injected with IL-6 and paw withdrawal thresholds were measured at 1 h, 3 h, 24 h, 48 h and 72 h post injection. For persistent sensitization experiments animals received an injection of PGE_2 _(100 ng) in the plantar surface of left hind paw in a volume of 25 μl 4 days following initial intraplantar injection. Following PGE_2 _injection, paw withdrawal thresholds were again measured at 1 h, 3 h and 24 h following the PGE_2 _injection.

### Plantar incision and behavioral testing

Prior to surgery all animals were assessed for paw withdrawal thresholds. A mouse model of incisional pain was used for this study [64]. A 5 mm longitudinal incision was made with a number 11 blade through skin, fascia and muscle of the plantar aspect of the hindpaw in isoflurane-anesthetized rats. Sham controls underwent the same procedure but without the incision. The skin was apposed with 2 sutures of 5 mm silk. Animals received intraplantar injection of resveratrol or vehicle around the incision at times indicated after incision. Animals were allowed to recover for 24 hrs and then paw withdrawal thresholds were measured at 24 hrs, 48 hrs, 72 hrs, 5, 7, 9, 11, and 13 days post-surgery. For persistent sensitization experiments, the animals received an intraplantar injection of PGE2 (100 ng/25 μl) 14 days following incision or sham procedures. The paw withdrawal thresholds were again measured at 1 h, 3 h and 24 h following the PGE2 injection.

### Primary neuronal cultures

Mouse trigeminal ganglia (TG) were excised aseptically and placed in Hank's Buffered Salt Solution (HBSS, Invitrogen) on ice. The ganglia were dissociated enzymatically with collagenase A (1 mg/ml, 25 min, Roche) and collagenase D (1 mg/ml, Roche) with papain (30 U/ml, Roche) for 20 min at 37°C. To eliminate debris 70 μm (BD) cell strainers were used. The dissociated cells were resuspended in DMEM/F12 (Invitrogen) containing 1× pen-strep (Invitrogen), 1× GlutaMax, 3 μg/ml 5-FDU (Sigma), 7 μg/ml uridine (Sigma), 50 ng/ml NGF (Millipore) and 10% fetal bovine serum (Hyclone). The cells were plated in 6-well plates (BD Falcon) and incubated at 37°C in a humidified 95% air/5% CO_2 _incubator. Cultures were maintained in resuspension media until time of treatment. For experiments where resveratrol treatments were done alone, cultures were maintained in the continuous presence of nerve growth factor (NGF) at a concentration of 50 ng/ml. NGF was excluded for IL-6 experiments. On day 5 the cells were washed in DMEM/F12 media for 30 mins and subsequently were treated as described in results.

### Western blotting

Protein was extracted from cells in lysis buffer (50 mM Tris HCl, 1% Triton X-100, 150 mM NaCl, and 1 mM EDTA at pH 7.4) containing protease and phosphatase inhibitor mixtures (Sigma) with an ultrasonicator on ice, and cleared of cellular debris and nuclei by centrifugation at 14,000 RCF for 15 min at 4°C. 15 μg of protein per well were loaded and separated by standard 7.5% or 10% SDS-PAGE. Proteins were transferred to Immobilon-P membranes (Millipore) and then blocked with 5% dry milk for 3 h at room temperature. The blots were incubated with primary antibody overnight at 4°C and detected the following day with donkey anti-rabbit antibody conjugated to horseradish peroxidase (Jackson Immunoresearch). Signal was detected by ECL on chemiluminescent films. Each phosphoprotein was normalized to the expression of the corresponding total protein on the same membrane. Densitometric analyses were performed using Image J software (NIH).

### 5' mRNA cap complex analysis

After the protein extraction, 50 μg protein was incubated with 7- methyl GTP Sepharose 4B beads (GE Healthcare) in the presence of 100 μM GTP for 2 h at 4°C. Unconjugated sepharose 4B beads were used for the negative controls. The beads were then pelleted and washed twice with lysis buffer. After a final centrifugation the pellet was suspended in 1× Laemmli Sample Buffer containing 5% v/v β-mercaptoethanol and eIF4E, eIF4G, eIF4A and 4EBP bound to the precipitated beads was analyzed by western blotting.

### Drugs and primary antibodies

Resveratrol was from Cayman Chemical; mouse 2.5S NGF was from Millipore; The following rabbit polyclonal antibodies were obtained from Cell Signaling: p-ERK (Thr202/Tyr204, cat# 4377), total ERK, p-eIF4E (Ser209, cat# 9741), total eIF4E, p-mTOR (Ser2448, cat# 2971), total mTOR, p-4EBP(Thr37/46, cat # 9459), total 4EBP, p-eIF4G (Ser1108, cat# 2441), total eIF4G, p-AKT (Ser473, cat# 4058), total AKT, GAPDH and eIF4A.

### Statistical Analysis and Data Presentation

Data are shown as means and the standard error of the mean (± SEM) of eight independent cell culture wells, 6 tissue samples (for in vivo Western blotting, eIF4F complex formation and nascent protein synthesis) or 6 animals (for behavioral studies). Graph plotting and statistical analysis used Graphpad Prism Version 5.03 (Graph Pad Software, Inc. San Diego, CA, USA). Statistical evaluation was performed by one- or two-way analysis of variance (ANOVA), followed by appropriate post-hoc tests. The a priori level of significance at 95% confidence level was considered at *p *< 0.05.

## Competing interests

The authors declare that they have no competing interests.

## Authors' contributions

TJP, GD, DVT, OKM and MNA conceived of the study and designed experiments, DVT, OKM, MNA, NQ, MDF and TJP performed experiments, DVT, OKM and TJP analyzed data, DVT, OKM and TJP wrote the manuscript. All authors read and approved the final manuscript.
